# Downregulation of RAI14 inhibits the proliferation and invasion of breast cancer cells

**DOI:** 10.7150/jca.34910

**Published:** 2019-10-18

**Authors:** Ming Gu, Wenhui Zheng, Mingdi Zhang, Xiaoshen Dong, Yan Zhao, Shuo Wang, Haiyang Jiang, Lu Liu, Xinyu Zheng

**Affiliations:** 1 Department of Breast Surgery, The First Affiliated Hospital of China Medical University, Shenyang, Liaoning 110001, People's Republic of China; 2 Department of anesthesiology, The Shengjing Hospital of China Medical University, Shenyang, Liaoning 110001, People's Republic of China; 3 Department of Breast Surgery, Obstetrics and Gynecology Hospital of Fudan University, Shanghai, 200011, People's Republic of China; 4 Lab 1, Cancer Institute, The First Affiliated Hospital of China Medical University, Shenyang, Liaoning 110001, People's Republic of China

**Keywords:** retinoic acid-induced 14, breast cancer, cell proliferation, migration, invasion

## Abstract

Retinoic acid-induced 14 (RAI14) is involved in the development of different tumor types, however, its expression and biological function in breast cancer are yet unknown. In the current study, we demonstrated that RAI14 was highly expressed in breast cancer. The high expression of RAI14 is positively correlated with the malignant progression of breast cancer and suggests a worse prognosis. Further, we found that knockdown RAI14 inhibits the proliferation, migration and invasion of breast cancer cells by regulating cell cycle and EMT through Akt/Cyclin D1, MMP2, MMP9 and ZEB1/E-cadhrin/Vimentin pathway. These findings revealed a novel function for RAI14 in breast cancer progression and suggest that RAI14 may become a promising diagnostic and therapeutic target for breast cancer.

## Introduction

Breast cancer is the most common female tumor worldwide. Due to the improvement of the level of diagnosis and treatment, breast cancer mortality rates have currently declined^1^. However, tumor invasion and metastasis remain the main cause of death in cancer patients. Indentifying the key proteins that promote the malignant progression of tumors and the development of new targeted drugs for breast cancer are important steps to improve the survival of cancer patients.

Retinoic acid-induced 14 (RAI14), also known as NORPEG, RAI13, is a novel protein-coding gene comprising six ankyrin repeats and two coil-coil domains 2. RAI14 was first discovered in liver and can be induced in human retinal pigment epithelial cells (ARPE-19) by all-trans retinoic acid 3. Studies have shown that RAI14 is expressed in many human tissues, especially in human placenta and testicular tissues 2, 4, and its function is closely related to the cytoskeleton. In recent years, more and more studies have found that RAI14 can be highly expressed in a variety of malignant tumors, including gastric cancer^5^-^7^, lung cancer^8^, ovarian cancer^9^ and prostate cancer^10^, and is positively correlated with the malignant progression of tumors. The high expression of RAI14 in these malignant tumors is significantly associated with the drug resistance response of tumor drugs and the proliferation and invasion of tumor cells. However, the expression and biological function of RAI14 in breast cancer have not been studied so far.

Our study aimed to analyze RAI14 expression in breast cancer tissue and its relevance to clinicopathological factors. Furthermore, we investigated the mechanism underlying the biological effects of RAI14 on breast cancer cells. Our results may provide a theoretical and experimental basis for the potential targeting of RAI14 in the diagnosis and treatment of breast cancer.

## Material and Methods

### Patients and specimens

Tissue samples were obtained from 137 female breast cancer patients, who had undergone breast surgery at the First Affiliated Hospital of China Medical University, between 2011 and 2014. All patients did not received any radiotherapy, chemotherapy, endocrine therapy or other treatment before surgery, while excluding patients with other malignant tumors, skin disease, epidermal ulcer, diabetes, and other diseases. The clinical stage was determined based on the World Health Organization classification. The status of ER, PR and HER2 were examined in the hospital. All patients have written informed consent for this study, which was approved by the regional ethics committee of China Medical University.

### Immunohistochemistry

The Immunohistochemical staining was performed on paraffin-embedded tissues according to the manufactuer's instructions of EnVision kit (MaiXin Biotech Co.,Fuzhou,China). The primary antibody was used rabbit anti-human RAI14 monoclonal antibody (1:150, Abcam, Cambridge, UK).The immunohistochemical scoring principle was according to the staining intensity (no signal=0, weak=1, moderate=2, high=3), and the percentage of staining cells (0%=0, 1%-10%=1, 11%-50%=2, 51%-80%=3, 81%-100%=4). The final score of 0-12 was based on multiplying the scores of intensity and percentage. The staining scores of RAI14 ≥4 was considered as high expression, <4 being regarded as low expression.

### Cell culture and plasmid transfection

Human breast cancer cell lines MCF7, MDA-MB-231, MDA-MB-453, T47D, and BT-549 were cultured in DMEM (Dulbecco's modified Eagle's medium) containing 10% FBS (fetal bovine serum) and 100 units/ml of penicillin/streptomycin at 37℃ in a 5%CO_2_ incubator. RAI14- and RAI14-RNAi-lentiviral vectors were purchased from Shanghai GeneChem Company (Shanghai, China). The RAI14 #1 sequence was 5'-AGAGTACGAGGAAATGAAA-3'; the RAI14 #2 sequence was 5'-AGACCTAAACCTTGTAGAT-3' and the shRNA control sequence was 5'-TTCTCCGAACGTGTCACGTtt-3'.

### Western blotting

Total protein was extracted in RIPA lysate with PMSF 1mM (Solarbio, Co. Ltd, Beijing, China), and quantified with BCA method. A total of 30 μg of protein was separated by 10% sodium dodecy1 sulfate-polyacrylaminde gel electrophoresis (SDS-PAGE), followed by transferred onto polyvinylidene fluoride (PVDF) membranes (Millipore, Billerica, MA, USA). The PVDF membranes were incubated with primary antibody: anti-RAI14 antibody (1:1000, Abcam, Cambridge, UK), p-Akt (1:1000, CST) , Akt (1:1000, CST), Cyclin D1 (1:1000, CST), MMP2 (1:1000, proteintech), MMP9 (1:1000, proteintech), E-cadherin (1:1000, CST), ZEB1 (1:1000, CST), Vimentin (1:1000, CST), at 4℃ overnight. After the membranes were incubated with horseradish peroxidase (HRP) conjugated secondary antibody and visualized by chemiluminescence ECL detection system (Bio-Rad).

### MTT assay

Cell proliferation was evaluated using MTT assay kit (Beyotime Biotechnology, Co., Ltd, Shanghai, China). The MTT solvent (5mg/ml) replaced medium to cells for 4h at 37℃, medium was removed and formed crystals were dissolved in 150 μl DMSO. The OD value was measured at 490 nm by enzyme immunoassay instrument.

### Colony formation assay

Cells were seeded in density 500 per well in 6 well plates. After 14 days cultured, colonies were fixed with 4% paraformaldehyde and stained with crystal violet. Colonies from 3 independent groups were counted and the data were presented as mean ± standard deviation (SD).

### Transwell assay

Transwell assay was used to evaluate cell migration and invasion. For migration assay, 4× 10^4^ cells were seeded in the upper in serum-free culture medium (200μl), and the lower chamber filled with complete medium. The cells were fixed with 4% paraformaldehyde and stained with gemsa 15min, after 24h incubation. The images were acquired under microscope and migrated cells were counted in 5 random fields. The method of invasion assay was similar to migration, but the upper chamber coated with matrigel (BD Bioscience, Bedford, MA, USA).

### Flow cytometry assay

Flow cytometry was carried out for the assessment cell cycle. Harvested cells at a density of 1× 10^6^ cells/ml were fixed with 75% ice-cold ethanol, and then washed in cold PBS. Before analysis, cells were incubated with bovine pancreatic RNase (2mg/ml, Sigma) for 30nin at 37℃, followed by treated with propidium iodide (PI, 20μg/ml, Sigma) for 20min. Cell cycle analysis was performed using a Flow Cytometry System (BD Bioscience, Bedford, MA, USA).

## Result

### RAI14 is overexpressed in breast cancers and correlated with clinicopathological features of breast cancer patients

**Figure 1:**
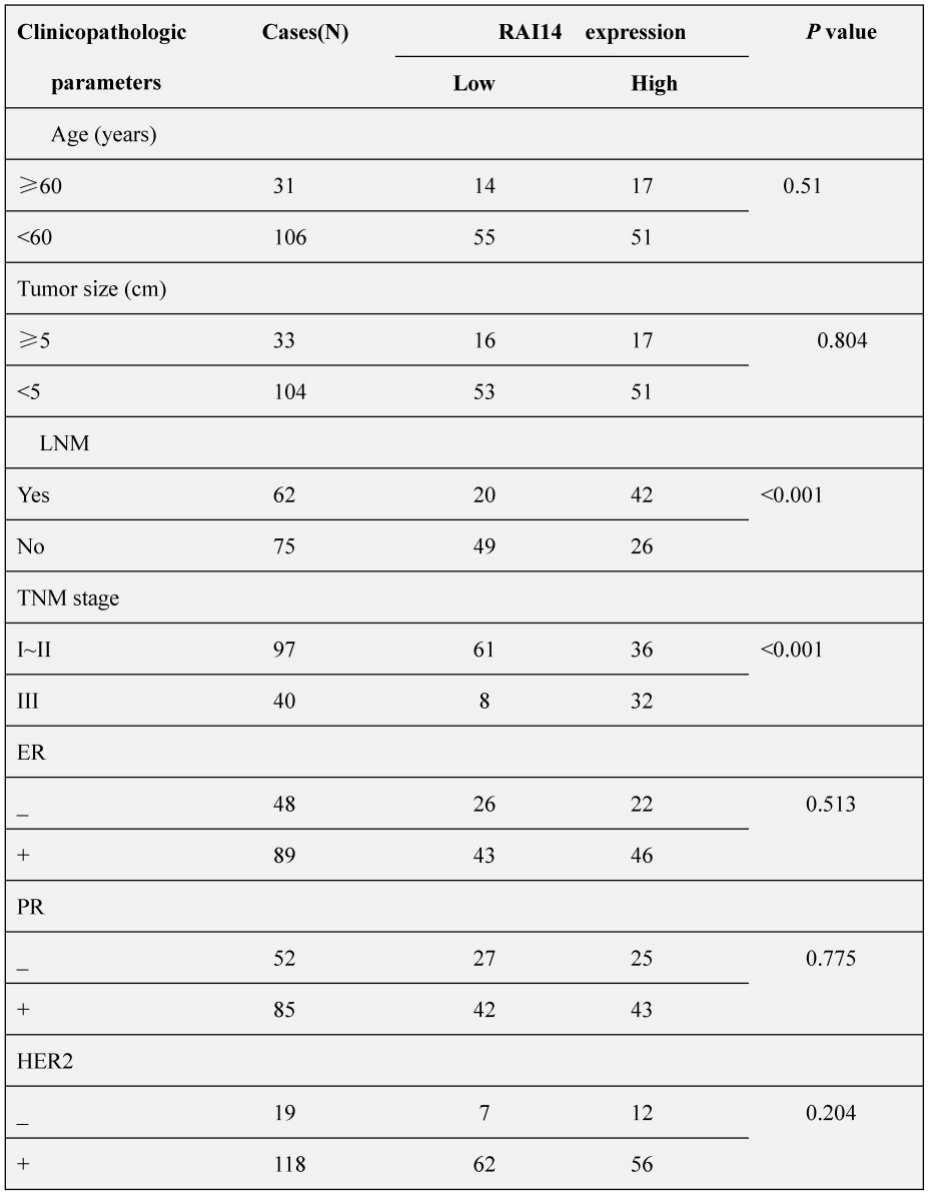

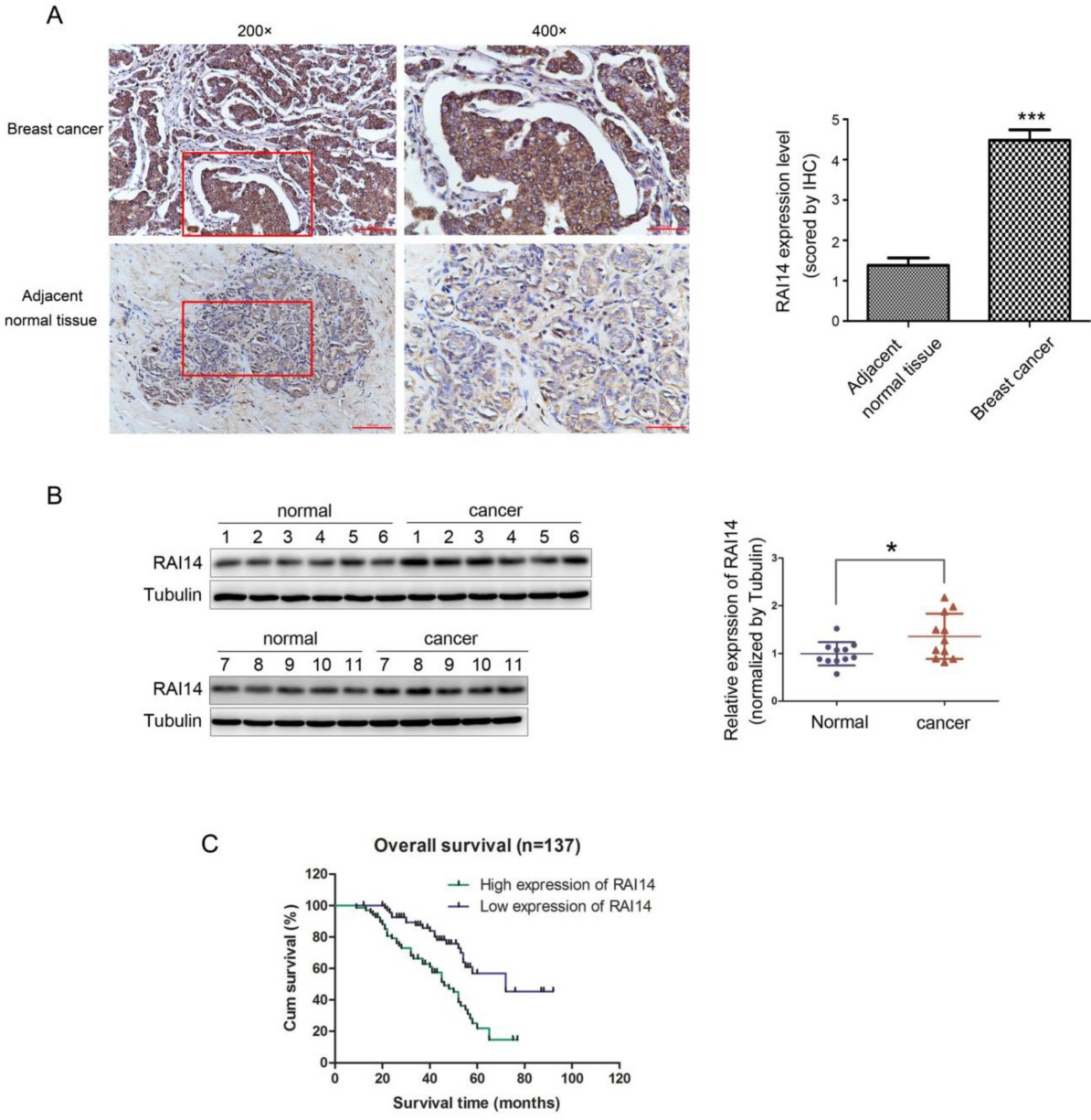
** Overexpression of RAI14 in breast cancer with worse prognosis.** (A) Immunohistochemistry results of RAI14 expression in paired breast cancer tissue samples. (B) Western blot analysis demonstrated the RAI14 expression in breast cancer tissues and normal tissues. RAI14 data visualized via scatter diagram. * P < 0.05. (C) Kaplen-Meir survival curves for 137 patients with breast cancer, grouped according to RAI14 expression.

We firstly determined the protein expression of RAI14 in 137 cases of breast carcinoma tissues and adjacent noncancerous tissues. As shown in Figure 1A, RAI14 protein was mainly localized at the cytoplasm of breast cancer cells, and the expression of RAI14 in tumor tissues was higher than that in adjacent normal tissues. Statistical analysis results showed that RAI14 expression was significantly correlated to lymph node metastasis (*p*<0.001) and advanced TNM stages (*p*<0.001), however, there was no difference between RAI14 expression and age (*p*=0.51), tumor size (*p*=0.804), estrogen receptor (ER) expression (*p*=0.513), progesterone receptor (PR) expression (*p*=0.775), and HER2 expression (*p*=0.204, Table 1). We also used western blotting assay to evaluate RAI14 protein in fresh tissues. The results showed that the expression level of RAI14 in breast cancer tissues was higher than that in normal tissues (Figure 1B). Furthermore, the Kaplan-Meier analysis showed that the survival time of patients with high RAI14 expression was shorter than those with low RAI14 expression (Figure 1C, *p*<0.01).

### Expression of RAI14 in human breast cancer cell lines and shRNA-mediated silence

**Figure 2:**
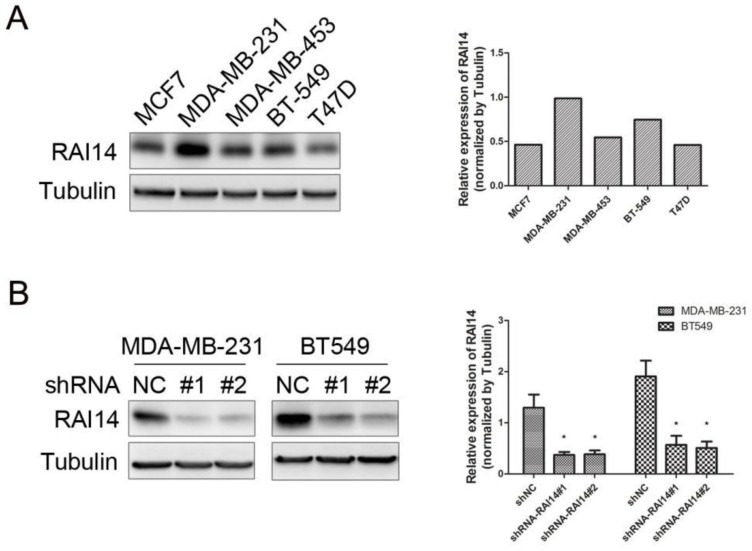
** Expression of RAI14 in breast cancer cell lines and knockdown of RAI14 by shRNA.** (A) Western blot showing the expression of RAI14 in 5 breast cancer cell lines. Tubulin served as protein loading control. (B) Stable RAI14 knockdown in MDA-MB-231 and BT549 cell lines was detected by immunoblot analysis. MDA-MB-231 and BT549 cells were stably transduced with two different lentiviral vectors, shRAI14 or the non-targeting control shRNA (shRNA NC). Quantitative data are shown. *P < 0.05 as compared to control.

We examined RAI14 expression in five breast cancer cell lines (MCF7, MDA-MB-231, MDA-MB-453, T47D, and BT-549) by Western blot. RAI14 was detected in all cell lines evaluated, and with MDA-MB-231 and BT549 cells expressing the highest level (Figure 2A). Therefore, MDA-MB-231 and BT549 cells were selected as the model for the subsequent function studies.

To study the function of RAI14 in MDA-MB-231 and BT549 cells, the RAI14 knockdown stable cell lines were used to analyze the silencing effect. As shown in Figure 2B, the level of RAI14 was significantly decreased in the shRNA-stable transfected cells compared to control.

### Knockdown of RAI14 expression inhibits the growth of breast cancer cells

**Figure 3:**
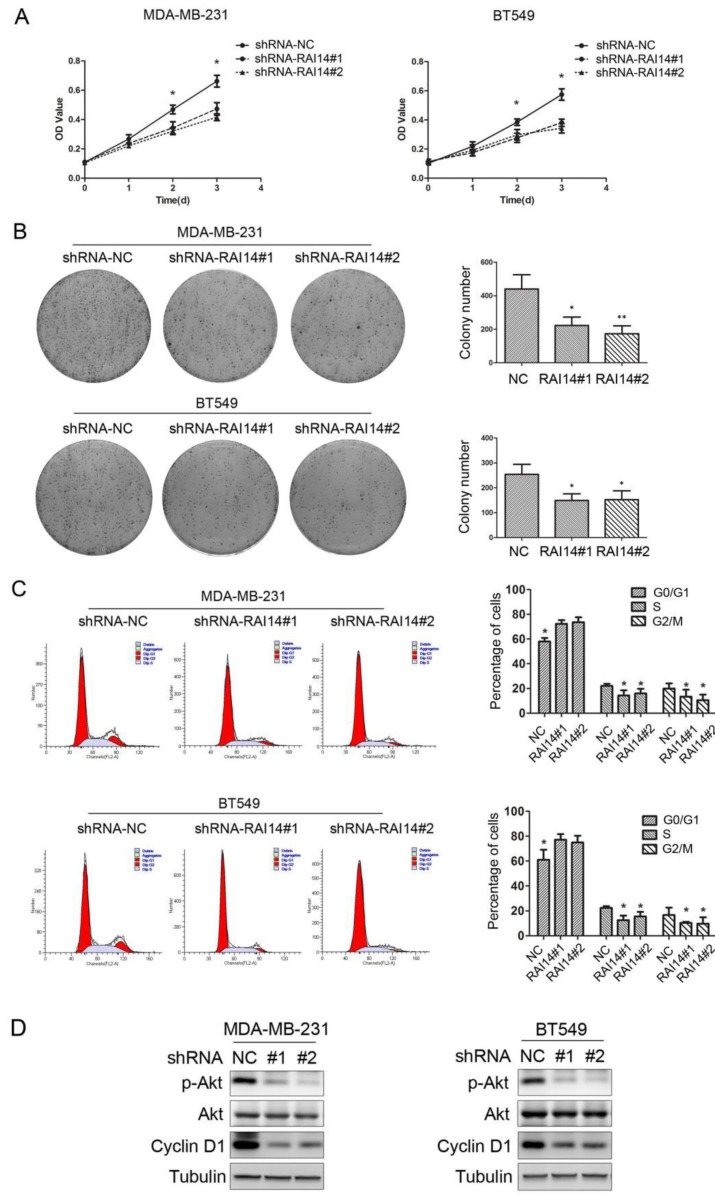
** Knockdown of RAI14 expression inhibits the proliferation of breast cancer cells.** (A)Proliferation was monitored by daily quantification of cell number for up to 3 days. * P < 0.05. (B) The colony forming assay showed that RAI14 knockdown inhibited cell growth in MDA-MB-231 and BT549 cells, * P < 0.05. (C) The cell cycle was suppressed after RAI14 knockdown. *P < 0.05. (D) The level of p-Akt and Cyclin D1 was decreased followed by knockdown RAI14, Tubulin served as protein loading control.

MTT assay was used to evaluate the growth of the cells in which RAI14 was knockdown. As shown in Figure 3A, RAI14 knockdown stable cells could inhibit proliferation of MDA-MB-231 and BT549 cells. To further test the effect of RAI14 knockdown on breast cancer cell growth, colony formation assay was performed. The results showed that RAI14 knockdown in MDA-MB-231 and BT549 cells formed significantly fewer colonies compared to MDA-MB-231 and BT549 cells (Figure 3B). It was observed that, when knockdown RAI14 expression in MDA-MB-231 and BT549 cells, the transition from the G1 to the S phase was significantly inhibited (Figure 3C). Our results revealed that AKT phosphorylation and the expression of Cyclin D1 were significantly inhibited in the shRAI14 compared with the shNC group (Figure 3D).

### Knockdown of RAI14 expression inhibits the migration and invasion of breast cancer cells

**Figure 4:**
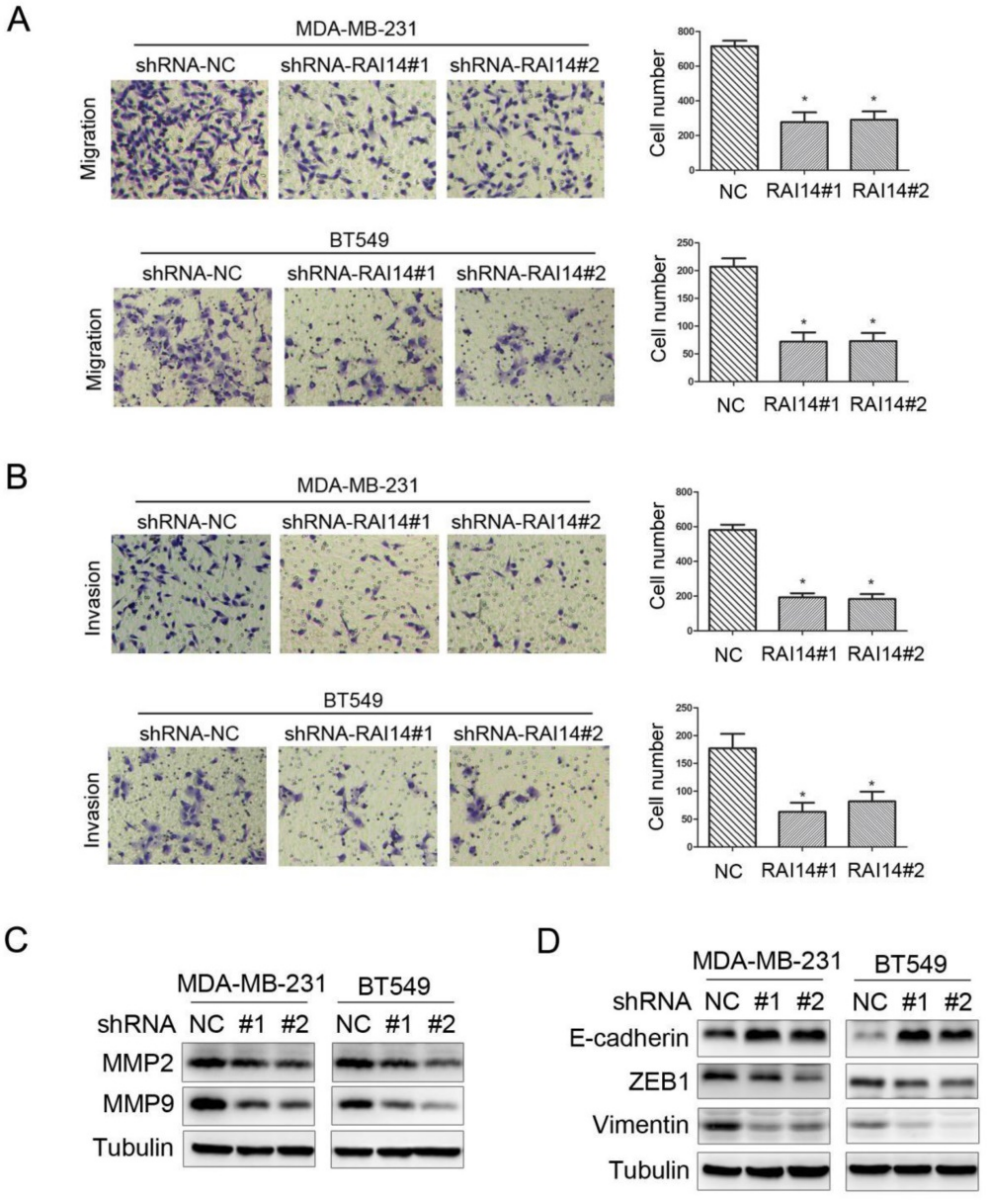
** Knockdown of RAI14 expression inhibits the migration and invasion of breast cancer cells.** (A and B) RAI14 knockdown retarded cellular migration (A) and invasion (B) in MDA-MB-231 and BT549 cell lines, respectively. The results are presented as a mean ± SD of three independent experiments. *P < 0.05. (C) Knockdown RAI14 inhibits the expression of MMP2 and MMP9, Tubulin served as protein loading control. (D) Knockdown RAI14 inhibits the expression of ZEB1, E-cadherin and Vimentin, Tubulin served as protein loading control.

Our migration assay revealed that the downregulation of RAI14 markedly suppressed MDA-MB-231 and BT549 cell migration (Figure 4A). Similarly, an invasion assay also indicated that RAI14 silencing significantly reduced cell invasion compared to control MDA-MB-231 and BT549 cells (Figure 4B), respectively. These results suggested that RAI14 silencing clearly inhibited breast cancer cell migration and invasion. Furthermore, western blotting analysis showed that knockdown of RAI14 inhibits the expression of MMP2 and MMP9 in breast cancer cells (Figure 4C). As indicated by the Western blot assay, the expression of the epithelial marker protein E-cadherin was increased, while the expression of the mesenchymal marker protein ZEB1 and vimentin were decreased in the shRAI14 group compared with the shNC group in both MDA-MB-231 and BT549 cells (Figure 4D). These results indicated that knockdown of RAI14 could inhibit the migration and invasion of breast cancer cells though affecting progression of EMT.

## Discussion

RAI14 was first discovered in liver and can be induced in human retinal pigment epithelial cells (ARPE-19) by all-trans retinoic acid 3. Nowadays, more and more research is now focusing on the role of RAI14 in tumors. In gastric cancer, RAI14 was highly expressed in cancer, and the high expression of RAI14 could be an independent predictor of poor prognosis in gastric cancer patients 6. RAI14 knockdown inhibited proliferation, migration and invasion and promoted apoptosis by downregulating the Akt pathway in gastric cancer cells, and RAB31 might be a downstream target gene of RAI14 7. In lung cancer, RAI14 was up-regulated in A549 and 31 of 71 patients. High expression of RAI14 could inhibit cell proliferation, indicating its potential as a new biomarker for lung adenocarcinoma 8. In ovarian cancer, NR2F2 regulates the expression of NEK2, RAI14, and multiple other genes involved in the cell cycle 9. In prostate cancer, RAI14 involved in the regulation of tumor cytoskeletal cell compartments 10.

In this study, we found that RAI14 is highly expressed in breast cancer cell lines and breast cancer tissues. Moreover, our clinicopathological data showed that RAI14 expression was significantly correlated to lymph node metastasis (*p*<0.001) and advanced TNM stages (*p*<0.001). We also found that downregulation of RAI14 can inhibit the proliferation, cell migration and invasion, which strongly suggests that RAI14 acts as an oncogene in breast cancer. Meanwhile, the RAI14 expression level was correlated with the prognosis of breast cancer patients, which indicated that RAI14 might be involved in progression of breast cancer.

Next, we want to explore how RAI14 affects cell proliferation, migration and invasion. AKT-Cyclin D1 pathway plays an important role in cell proliferation 11, 12. Previous study has reported that RAI14 knockdown significantly reduced the level of the phosphorylated form p-Akt in gastric cancer cells. Meanwhile, the expression of its downstream protein Cyclin D1 was also decreased in the RAI14 deficiency cells. We also found that down-regulation of RAI14 can inhibit the phosphorylation of Akt and the expression of Cyclin D1 in breast cancer cells, which is consistent with previous reports 7.

Degradation of the basal membrane is a necessary step in most cancers, and the MMPs have played an important role in this process 13, 14. In our study, we found that RAI14 knockdown reduced the expression levels of MMP2 and MMP9. In breast cancer, EMT has been defined as a critical component of the metastatic process 15, 16. Once EMT occurs, cancer cells acquire phenotypes that are more likely to invade 16. The occurrence of EMT is believed to change the expression of genes whose products play a key role in maintaining epithelial status, such as e-cadherin 17, and this inhibition occurs at the transcription level through the action of EMT transcription factors, such as ZEB1^18^. In our study, we found that RAI14 knockdown increased the expression of epithelial marker e-cadherin and decreased the expression of mesenchymal marker vimentin. Meanwhile, the expression of EMT transcription factor ZEB1 is reduced when knockdown RAI14 suppresses EMT. These results suggest that RAI14 silencing reduces the migration and invasion of breast cancer cells by inhibiting EMT.

Our study demonstrated that RAI14 was highly expressed in breast cancer. The high expression of RAI14 is positively correlated with the malignant progression of breast cancer and suggests a worse prognosis. Further, we found that RAI14 affects the proliferation, migration and invasion of breast cancer cells by regulating cell cycle and EMT. Our study elucidates the effect of RAI14 on the development of breast cancer cells and might provide a novel target for cancer therapy.

## Figures and Tables

**Table 1:** Correlation between RAI14 and clinicopathologic parameters of breast cancer patients

## References

[1] Siegel RL, Miller KD, Jemal A (2017). Cancer Statistics. CA Cancer J Clin.

[2] Peng YF (2000). Ankycorbin: a novel actin cytoskeleton-associated protein. Genes Cells.

[3] Kutty RK (2001). Molecular characterization and developmental expression of NORPEG, a novel gene induced by retinoic acid. J Biol Chem.

[4] Yuan W (2005). Expression of a novel alternative transcript of the novel retinal pigment epithelial cell gene NORPEG in human testes. Asian J Androl.

[5] Zhou J (2015). An integrative approach identified genes associated with drug response in gastric cancer. Carcinogenesis.

[6] He XY (2018). High Expression of Retinoic Acid Induced 14 (RAI14) in Gastric Cancer and Its Prognostic Value. Med Sci Monit.

[7] Chen C (2018). Knockdown of RAI14 suppresses the progression of gastric cancer. Onco Targets Ther.

[8] Yuan C (2017). Super enhancer associated RAI14 is a new potential biomarker in lung adenocarcinoma. Oncotarget.

[9] Hawkins SM (2013). Expression and functional pathway analysis of nuclear receptor NR2F2 in ovarian cancer. J Clin Endocrinol Metab.

[10] Paez AV (2016). Heme oxygenase-1 in the forefront of a multi-molecular network that governs cell-cell contacts and filopodia-induced zippering in prostate cancer. Cell Death Dis.

[11] Walker JL, Assoian RK (2005). Integrin-dependent signal transduction regulating cyclin D1 expression and G1 phase cell cycle progression. Cancer Metastasis Rev.

[12] Yakes FM (2002). Herceptin-induced inhibition of phosphatidylinositol-3 kinase and Akt Is required for antibody-mediated effects on p27, cyclin D1, and antitumor action. Cancer Res.

[13] Javadian M (2018). The role of microRNAs regulating the expression of matrix metalloproteinases (MMPs) in breast cancer development, progression, and metastasis.

[14] Chabottaux V, Noel A (2007). Breast cancer progression: insights into multifaceted matrix metalloproteinases. Clin Exp Metastasis.

[15] Hong D (2018). Epithelial-to-mesenchymal transition and cancer stem cells contribute to breast cancer heterogeneity. J Cell Physiol.

[16] Thiery JP (2009). Epithelial-mesenchymal transitions in development and disease. Cell.

[17] Aiello NM (2018). EMT Subtype Influences Epithelial Plasticity and Mode of Cell Migration. Dev Cell.

[18] Nieto MA (2016). Emt:2016. Cell.

